# Within-food-group optimization improves nutritional adequacy, sustainability, and acceptability of modeled diets

**DOI:** 10.3389/fnut.2025.1648055

**Published:** 2025-09-30

**Authors:** Dominique van Wonderen, Johanna C. Gerdessen, Sander Biesbroek, Alida Melse-Boonstra

**Affiliations:** ^1^Wageningen Social & Economic Research, Wageningen University & Research, Wageningen, Netherlands; ^2^Group Operations Research & Logistics, Wageningen University & Research, Wageningen, Netherlands; ^3^Division of Human Nutrition and Health, Wageningen University & Research, Wageningen, Netherlands

**Keywords:** consumer acceptability, dietary change, diet modeling, diet optimization, food substitution

## Abstract

**Introduction:**

Food production is a major contributor to global greenhouse gas emissions (GHGE). To mitigate this impact, researchers have developed methods for designing healthy and sustainable diets by modifying existing consumption patterns through dietary changes between food groups. However, the nutrient and emission profiles within these food groups can differ greatly. The aim of this study was to investigate the extent to which the nutritional adequacy, sustainability, and acceptability of diets can be improved through dietary changes within food groups.

**Methods:**

To analyze the potential of within-food-group optimization, we investigated several diet modeling strategies and scenarios to optimize nutrient intake while minimizing GHGE and dietary change. The diets used as input for the diet model were derived from the U.S. National Health and Nutrition Examination Survey (NHANES) 2017–2018 consumption dataset.

**Results:**

By adjusting food quantities only within food groups, macro- and micronutrient recommendations could be met while achieving a 15 to 36% reduction in GHGE. When foods were optimized both within- and between food groups, only half the dietary change (23%) was required to achieve a 30% GHGE reduction, compared to optimizing between food groups alone (44%). This may improve consumer acceptance, assuming smaller dietary shifts are perceived as more acceptable.

**Conclusion:**

Within-food-group optimization increases opportunities to improve the nutritional adequacy, sustainability, and acceptability of diets.

## 1 Introduction

Food production for human consumption is estimated to contribute one-third of global greenhouse gas emissions (GHGE) caused by human activities (1). To reduce this footprint, various methodologies have been developed to design diets that are both environmentally and nutritionally sustainable. This research area is commonly referred to as diet modeling or diet optimization. Initial studies focused on defining nutritionally adequate diets within set cost limits, but from around 2010 onward, they began to incorporate environmental considerations (2–5).

Multiple studies have shown that substantial dietary changes are necessary to improve the sustainability of diets, though the precise extent varies between studies (Table 1). For example, Vieux et al. (9) examined the changes in food consumption needed to reduce GHGE in several European countries while complying with nutrient guidelines. Based on their results, it was computed that achieving a 30% reduction in GHGE required food quantity changes ranging from 40 to 65%. Similarly, it could be calculated from the study by Rocabois et al. (13) that a 30% reduction in GHGE would involve food quantity changes of up to 69% in the French diet. Other studies have reported a lower level of required dietary change. For instance, Nordman et al. (18) found that a 30% change in the Danish diet could achieve a 31% reduction in GHGE.

**Table 1:** Summary of diet modeling studies showing the trade-off between greenhouse gas emissions (GHGE) and total dietary change, as well as detailing the number of food groups used and the number of individual food items aggregated within those groups.

Study	Country^a^	Trade-off^b^^,^^c^		Number of food groups^d^	Number of individual food items
		GHGE reduction (%)	Dietary change (%)^e^		
Green et al. (6)	UK	30–60	68–94	148	2,980
Horgan et al. (7)	UK	15	50^f^^,^^g^	134	1,491
Perignon et al. (2)	FR	10–60	5–50^g^	402	1,342
Gazan et al. (8)	FR	—	—	212	1,342
Vieux et al. (9)	FR, UK, IT, FI, SE	30	40–65	151	1,708–4,079
Reynolds et al. (10)	UK	57	54	101	653
Mariotti et al. (11)	FR	—	—	32	2,800
Dussiot et al. (12)	FR	—	—	45	1,533
Rocabois et al. (13)	FR	30	69	207	1,342
Tompa et al. (14)	HU	—	—	35	857
Heerschop et al. (15)	NL	16	33	28	2,389
Verly et al. (16)	BR	—	—	85	1,591
Fu et al. (17)	CN	—	—	11	—
Nordman et al. (18)	DK	31	30	50	434
Bashiri et al. (19)	EE	—	—	14	74
Fouillet et al. (20)	FR	—	—	45	1,533
Kesse-Guyot et al. (21)	FR	77	110	47	265

a Country of the consumption data set used for modeling.

b Results on GHGE reductions and total dietary change should be interpreted with caution across studies, as different modeling approaches were applied, limiting direct comparability.

c A “—” indicates that GHGE reductions or total dietary change were not reported or could not be calculated from the study’s results.

d Some studies refer to food groups as food items. For example, Vieux et al. (9) model diets using 151 food items such as root vegetables and citrus fruits. In this study, we classify these as food groups, with items like carrots and oranges representing individual foods within those groups.

e See Supplementary Method 1 for the calculation of total dietary change.

f Average dietary change calculated at the food group level, not at the total diet level.

g Complete removal or addition of food groups is not included in this number.

The differences in modeling results regarding GHGE reductions and required dietary changes can be explained by several factors. These include variations in the current consumption patterns of the target group under investigation, as well as in the GHGE and nutrient content of available foods, which may differ between countries. Results are also influenced by modeling decisions, such as the nutrient constraints applied, food quantity limits set to ensure the acceptability of modeled diets, and the level of detail at which foods are represented.

Diet modeling studies often model dietary changes at the level of food groups or subgroups, both referred to as food groups in this study. The number of food groups considered varies widely, ranging from 11 (17) to 402 (2) (Table 1). When modeling at food group level, quantities of food groups can be adjusted, but the distribution of individual food items within each group remains unchanged, or an average food is used as proxy. For instance, while the overall quantity of the food group “vegetables” can be increased, it is not possible to adjust the distribution of specific food items, such as increasing “carrots” while decreasing “cucumber.”

**Figure 1:**
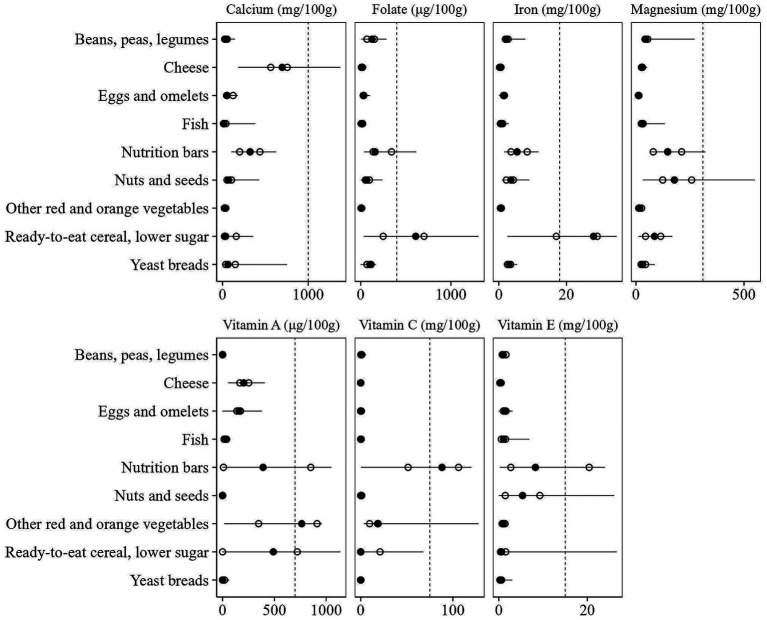
Variation in nutrient content within a selection of What We Eat in America (WWEIA) food subgroups that display considerable differences. The horizontal line shows the range of variation, with the black dot marking the average content and the white dots representing the 25th and 75th percentiles. The vertical line indicates the Recommended Daily Allowance (RDA).

**Figure 2:**
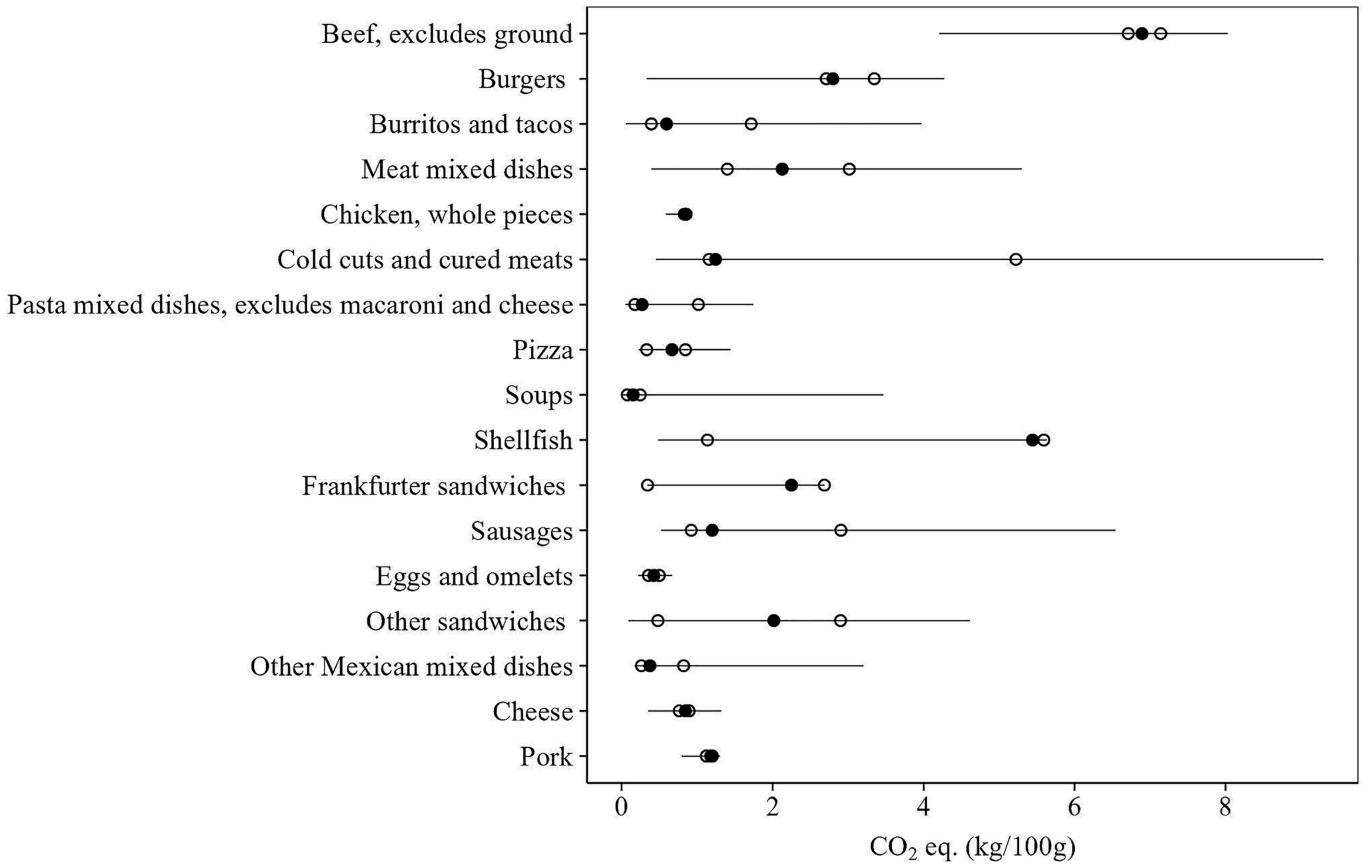
Variation in greenhouse gas emissions across the What We Eat in America (WWEIA) food subgroups that contribute most to observed diet-related CO_2_ eq. emissions, limited to groups with three or more food items. The horizontal line shows the range of variation, with the black dot marking the average emission and the white dots representing the 25th and 75th percentiles.

However, when modeling at food group level, the variability in nutrient composition (Figure 1) and GHGE profiles (Figure 2) within food groups (22) is not taken into account, leaving opportunities to further improve the nutritional adequacy and sustainability of diets by optimizing foods within those food groups (23). In addition, within-food-group optimization could improve the acceptance of generated diets for two reasons. First, foods within food groups are typically more similar than those between food groups, which may make such substitutions more preferable (24, 25). Second, consumer acceptance is often linked to the extent of dietary change, with smaller changes generally considered more achievable (4, 26). By allowing changes within food groups, rather than only between food groups, the total dietary change required could be reduced, thereby increasing acceptance.

The impact of whether foods are optimized within food groups on the nutritional adequacy, environmental sustainability, and acceptability of diets has not yet been quantified. Accordingly, this study aims to demonstrate the potential benefits of within-group optimization, which may help future studies determine the most suitable level at which to model foods. To achieve this, we developed a diet model that optimizes nutrient intake while minimizing GHGE and dietary change by adjusting food quantities reported in the U.S. National Health and Nutrition Examination Survey 2017–2018 (NHANES). By exploring different modeling strategies, we evaluated and compared the performance of between-food-group and within-food-group optimization, along with the trade-offs among nutritional adequacy, environmental sustainability, and acceptability.

## 2 Materials and methods

To demonstrate the impact of within-food-group optimization on the nutritional adequacy, environmental sustainability, and acceptability of diets, we started from observed consumption data and applied various diet modeling strategies to optimize nutrient intake while minimizing greenhouse gas emissions (GHGE) and dietary changes for both females and males.

### 2.1 Data

#### 2.1.1 Observed diet

U.S. food consumption data, comprising two 24-h dietary recalls, were retrieved from NHANES 2017–2018 (27), and corresponding nutrient intakes from the Food and Nutrient Database for Dietary Studies (FNDDS) 2017–2018 (28). For the purpose of this study, which is to compare methodological approaches, we focused on a single target group: adults aged 18 to 65, as they represent a relatively homogeneous group in terms of nutritional needs. Individuals were excluded if they reported a low energy intake (less than 1,200 kcal for women and 1,800 kcal for men) or a high energy intake (more than 3,000 kcal for women and 3,600 kcal for men). This left 1,738 female respondents and 1,428 male respondents, with 3,166 respondents in total. The consumption data were then summarized by calculating the average daily intake per food item (g/day) separately for females and males, which is referred to as the observed diets.

#### 2.1.2 Food group classification

The food group classifications used for diet modeling were based on the What We Eat in America (WWEIA) (29) and FNDDS (28) subgroup classifications, comprising 153 and 46 groups, respectively. In addition, a custom food group classification was created using the method as described by Perignon et al. (2), consisting of 345 groups (Supplementary Method 2). Separate model runs were conducted for each food group classification.

Of the 4,257 reported unique food items, the intake of 2,734 food items was considered for optimization. Foods excluded from optimization, i.e., for which quantities were kept identical to those in the observed diets, included items classified as “other” according to the WWEIA food group classification (e.g., nutritional powder mix and raw oats). Furthermore, food items consumed three times or less were excluded from optimization. An overview of the included food items and their corresponding food groups is provided in “Food Group Classifications” of Supplementary File.

#### 2.1.3 Greenhouse gas emissions

GHGE of NHANES (composite) foods, expressed in CO_2_ equivalents, were estimated using GHGE data for corresponding primary food products from the dataFIELD database and associated loss factors from the Loss-Adjusted Food Availability (LAFA) database (30). LAFA provides the percentage of food weight lost throughout the supply chain (from farm to retail) as well as losses at the consumer level (e.g., inedible portions, cooking losses, and uneaten food). GHGE for each NHANES food was calculated as:


$$\begin{array}{l} \text{GHGE}=\sum_{i}{\text{Weight}}_{i}\cdot {\text{GHGE}}_{i}\cdot \frac{100}{100-\text{Food loss}\;{\left(\%\right)}_{i}}\Rightarrow \\ \left(\frac{{\mathrm{CO}}_{2}\mathrm{eq}.}{100\;g})=(\frac{g}{100\;g})\cdot (\frac{{\mathrm{CO}}_{2}\mathrm{eq}.}{g}\right) \end{array}$$

where i represents the primary food products of the NHANES food. The weights of primary food products for each NHANES food were based on the Food Commodity Intake Database (FCID) (19), extended by Fouillet et al. (20) for more recent NHANES cycles (2011–2018). Both the weight and GHGE data align with FCID’s classification of primary food products. For the loss factors, LAFA food descriptions were mapped to FCID food codes by Conrad et al. (31). Using this approach, the calculated GHGE for the observed diets was 5.1 kg CO_2_ equivalents per day for females and 7.9 kg CO_2_ equivalents per day for males.

### 2.2 Diet modeling

The diet model optimized nutrient intake while minimizing GHGE and dietary change by adjusting food quantities. Different strategies for achieving this were explored to compare the performance of between- and within-food-group optimization, as well as to compute the trade-offs between nutritional adequacy, environmental sustainability, and acceptability. Full model definitions are provided in Supplementary Tables 1–3, with the applied nutritional guidelines outlined in Supplementary Table 4.

#### 2.2.1 Within-food-group optimization

In the first modeling experiment, we examined the potential to improve the nutritional adequacy and GHGE of observed diets by adjusting food item quantities within food groups only. The model permitted changes only to the distribution of food items within each group, keeping the overall quantity of each food group similar to the observed diet. In the objective function of the model, the highest weight was assigned to minimizing the largest deviation from recommended macro- and micronutrient intake levels (Recommended Daily Allowances; RDA), then GHGE reduction, and finally, minimizing dietary change. A simplified version of the objective function is given by:


$$\min\{D^{\text{macro}}+D^{\mathrm{rda}}+\varepsilon_{1}\cdot E+\varepsilon_{2}\cdot C^{\text{within}}\}$$

where $D^{\text{macro}}$ and $D^{\mathrm{rda}}$ represent deviations from macronutrient and micronutrient (RDA) guidelines, E represents GHGE, $C^{\text{within}}$ represents within-food-group changes, and $\varepsilon_{1}$ and $\varepsilon_{2}$ are small weighting values such that $\varepsilon_{1}>\varepsilon_{2}$ . The full formulation is provided in Equation 30 of Supplementary Table 3.

Furthermore, we evaluated model outcomes using three different food group classifications (Perignon with 345 groups, WWEIA with 153 groups, and FNDDS with 46 groups) across varying levels of within-food-group changes allowed (0, 25, 50, 75, and 100%). When no change was allowed (0% change), the diet remained identical to the observed diet. In the least restrictive scenario (100% change), all food item quantities within each food group could be adjusted freely. For intermediate scenarios, we constrained the absolute change within each food group to x% or less. Additionally, no food item could exceed the quantity of the highest-consumed item in that food group. Note that these upper bounds for food item quantities were based on daily average intakes from the consumption dataset and do not reflect the highest intakes reported by individuals. The unrestricted scenario may favor a narrow selection of the healthiest and most sustainable foods, whereas the restricted scenarios lead to more diverse and balanced diets, which we assumed are more acceptable. This allowed us to examine the trade-off between acceptability, nutritional adequacy, and sustainability.

#### 2.2.2 Lowered nutrient goals

In the second modeling experiment, which builds on the first, we investigated to what extent lowering nutrient goals can further reduce GHGE. This provides a sharper picture on the trade-off between nutritional adequacy and sustainability. Here, macronutrient bounds were extended by 20% (e.g., the protein lower and upper bounds were relaxed from 10–35 E% to 8–42 E%), and micronutrient goals were lowered from the RDA to the Estimated Average Requirements (EAR):


$$\min\{D^{\text{macro}\pm 20\%}+D^{\mathrm{ear}}+\varepsilon_{1}\cdot E+\varepsilon_{2}\cdot C^{\text{within}}\}$$

For the full formulation, see Equation 31 of Supplementary Table 3.

#### 2.2.3 Between-food-group vs. between-and-within-food-group optimization

In the last modeling experiment, we investigated the minimal amount of dietary change required to achieve at least a 30% reduction in GHGE while complying with macronutrient and micronutrient (RDA) guidelines:


$$\min\{C^{\text{total}}+\varepsilon \cdot E\}$$

where $C^{\text{total}}$ represents total dietary change and $D^{\text{macro}}=D^{\mathrm{rda}}=0$ (see Equation 37 of Supplementary Table 3).

We compared the required dietary change under two scenarios: one where only total food group quantities may be adjusted while distribution of food items within each group remains fixed (between-food-group optimization), and another where food item quantities can be changed without restriction (between-and-within-food-group optimization). This comparison demonstrates how within-food-group optimization can improve the acceptability of designed diets.

Acceptability was evaluated based on total dietary change (Supplementary Method 1) and food diversity. We assumed that diets requiring smaller deviations from observed consumption patterns are more acceptable (4, 26). Food diversity was measured by the number of unique food items and the average share of the most prevalent food item in each food group, where diets with a higher number of unique food items and a lower average share were considered more diverse and balanced, and therefore more acceptable (32, 33).

## 3 Results

To demonstrate the added value of within-food-group optimization on the nutritional adequacy, environmental sustainability, and acceptability of diets, we conducted various modeling experiments, for which the results are displayed below.

### 3.1 Within-food-group optimization

**Figure 3:**
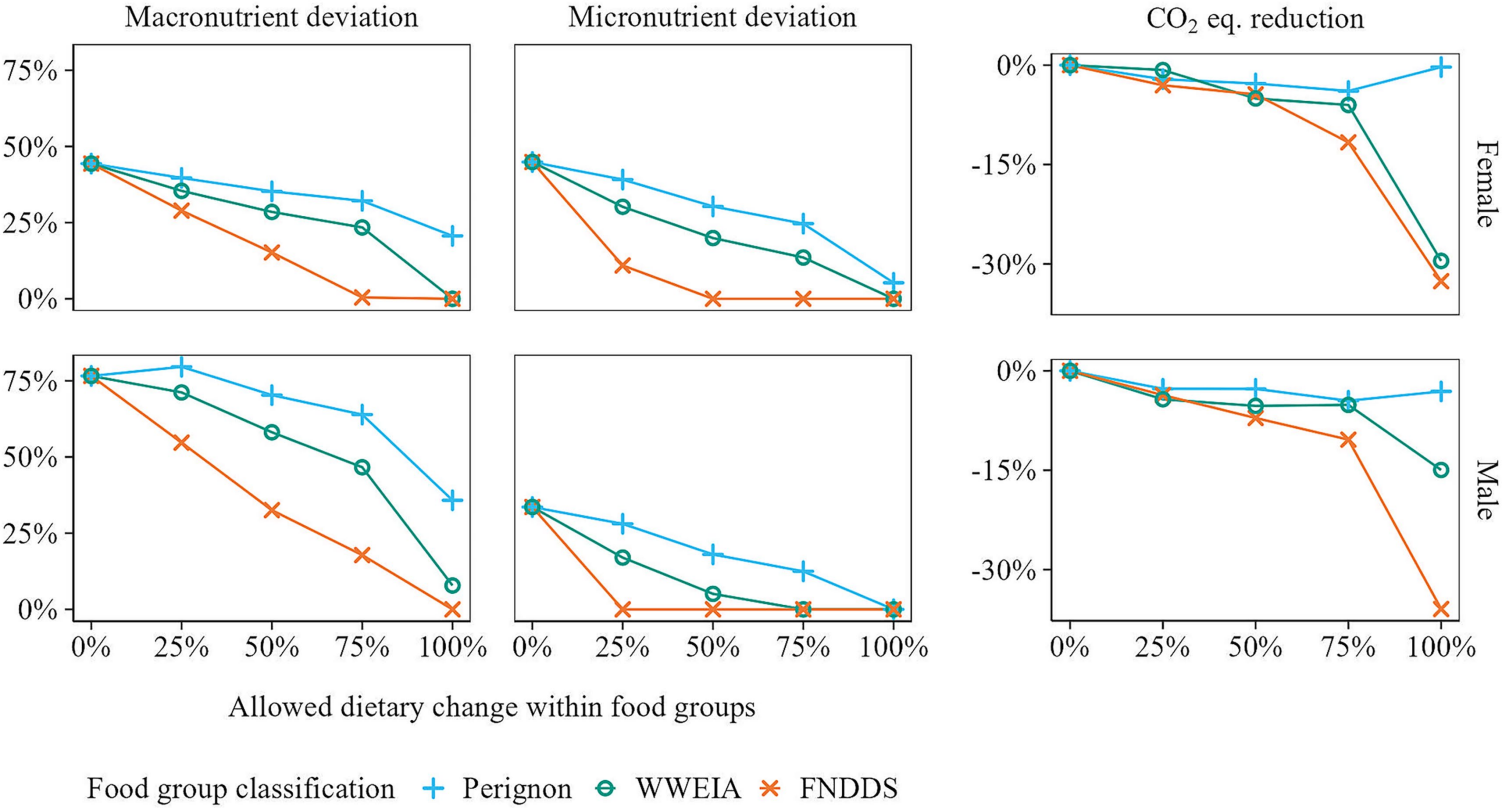
Deviations from dietary guidelines for the most limiting macro- and micronutrient, along with reductions in greenhouse gas emissions (GHGE), for observed and optimized diets in which food item quantities were adjusted within food groups only. As the diet model prioritizes optimizing nutritional adequacy over sustainability (Supplementary Method 3), a reduction in macro- and micronutrient deviations may come at the cost of increased GHGE, as demonstrated by the scenario where a dietary change of 100% was allowed for the Perignon food group classification.

In the first modeling experiment, we examined to what extent the nutritional adequacy and GHGE of observed diets could be improved by adjusting food item quantities within food groups only. Figure 3 shows how deviations from macro- and micronutrient guidelines and GHGE were reduced, with greater reductions observed when changes within groups were larger. When no restrictions were placed on the magnitude of change (allowed within food group change of 100% and no constraint on the maximum quantity of food items), deviations from dietary guidelines were either absent or negligible, and GHGE was reduced by 15 to 36%, when using the WWEIA or FNDDS food group classification. However, when using the Perignon classification, the largest deviations were still 36% for macronutrients and 1% for micronutrients, with a maximum GHGE reduction of just 3%.

**Figure 4:**
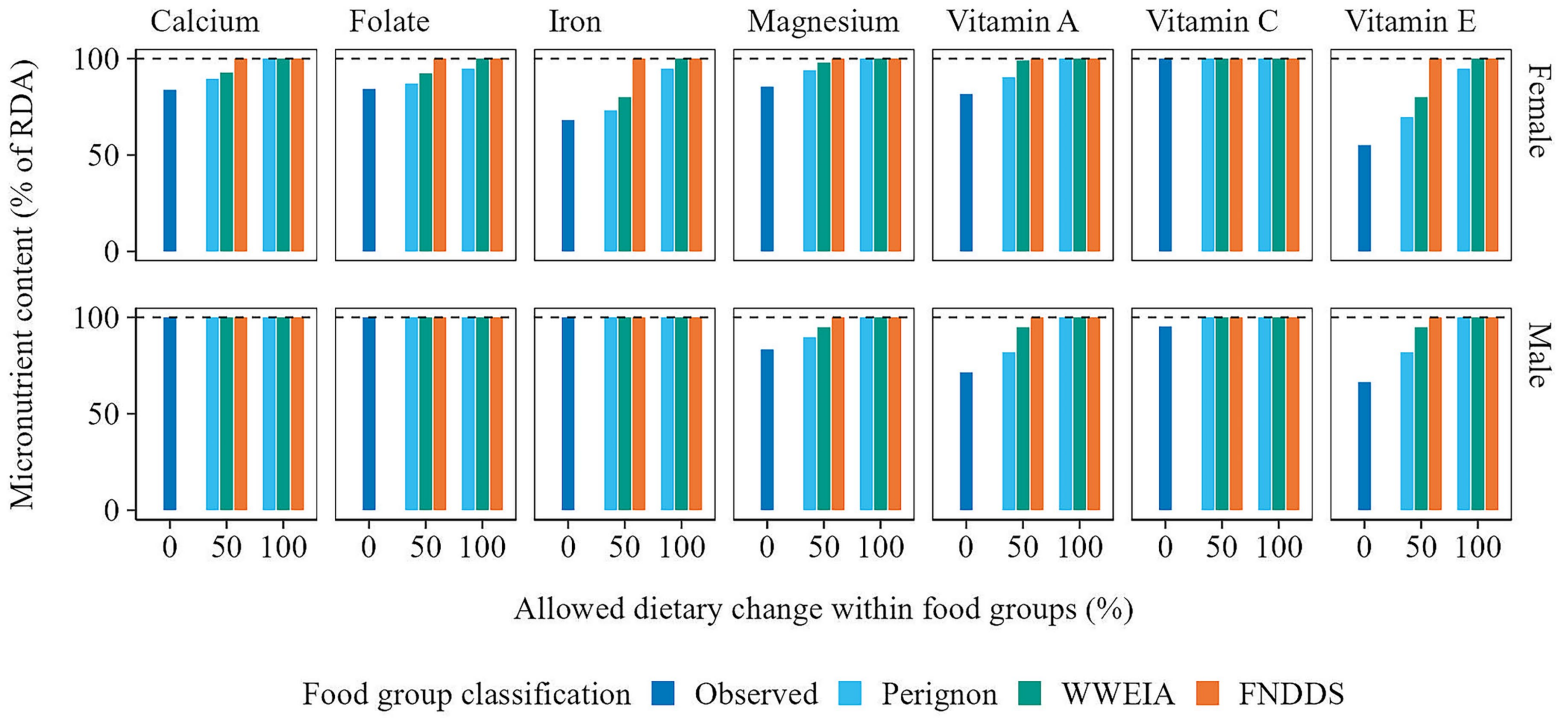
Average micronutrient content of the observed and optimized diets in which food item quantities were adjusted within food groups only. The micronutrient content is presented as a percentage of the Recommended Daily Allowance (RDA). Note that only micronutrients with values below the RDA are displayed. Additionally, nutrient intake is capped at 100%.

**Figure 5:**
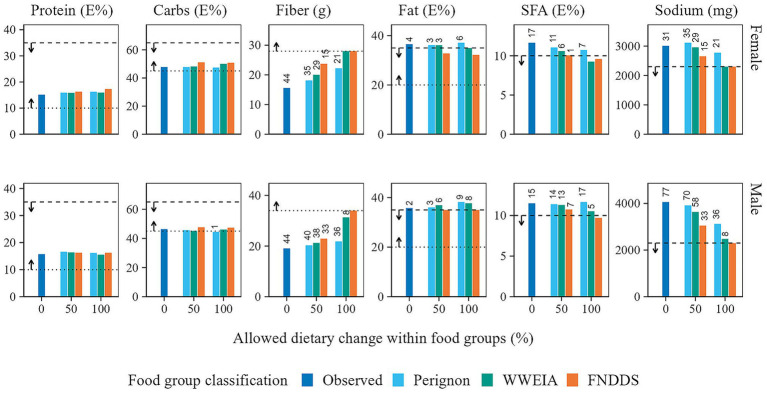
Macronutrient and sodium content of the observed and optimized diets in which food item quantities were adjusted within food groups only. For protein, carbohydrate, total fat, and saturated fatty acids (SFA), the nutrient content is expressed as a percentage of energy intake. The dotted and dashed horizontal lines display the lower and upper bounds of recommended intake, respectively. The labels above the bars display the deviation from dietary guidelines (%).

Figures 4, 5 offer a closer look at the micro- and macro-nutrient content of observed and optimized diets. For females, the average micronutrient content of the observed diets was below the RDA for calcium, folate, iron, magnesium, vitamin A, and vitamin E. Increasing the intake of iron and vitamin E, followed by calcium and folate, proved to be the most difficult, as shown by the Perignon and WWEIA food group classifications where the allowed within food group change was 50%. For males, the bottleneck nutrients were magnesium, vitamin A, and vitamin E. In terms of macronutrient and sodium content, the observed diets for both females and males failed to meet the dietary guidelines for fiber, total fat, saturated fatty acids (SFA), and sodium. Optimizing these nutrients proved challenging, with only the scenarios allowing 100% within food group changes, using the WWEIA and FNDDS classifications, meeting or nearly meeting the dietary guidelines.

**Figure 6:**
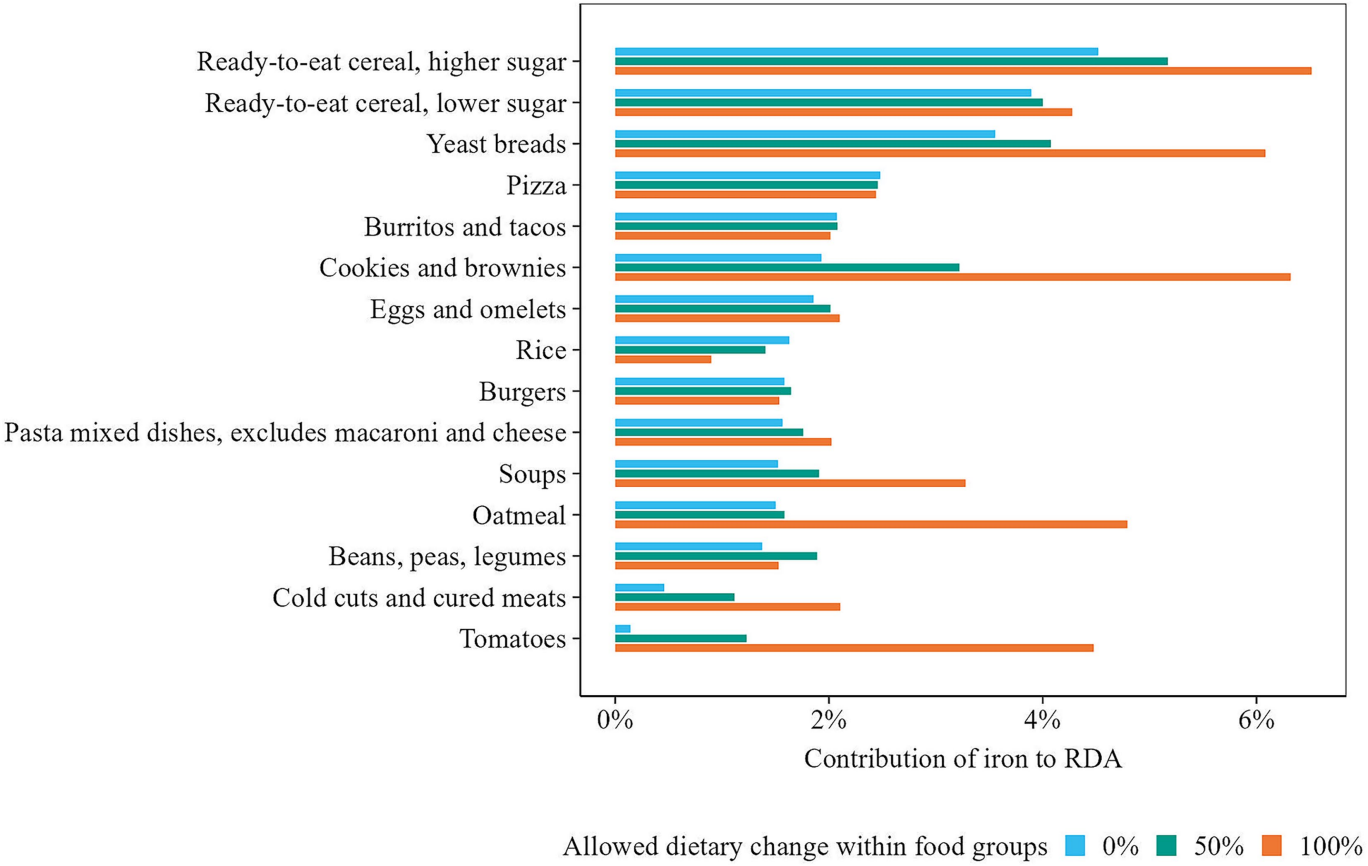
Iron content of WWEIA groups contributing most to iron intake in observed and optimized diets for females, presented as a percentage of the Recommended Daily Allowance (RDA).

**Figure 7:**
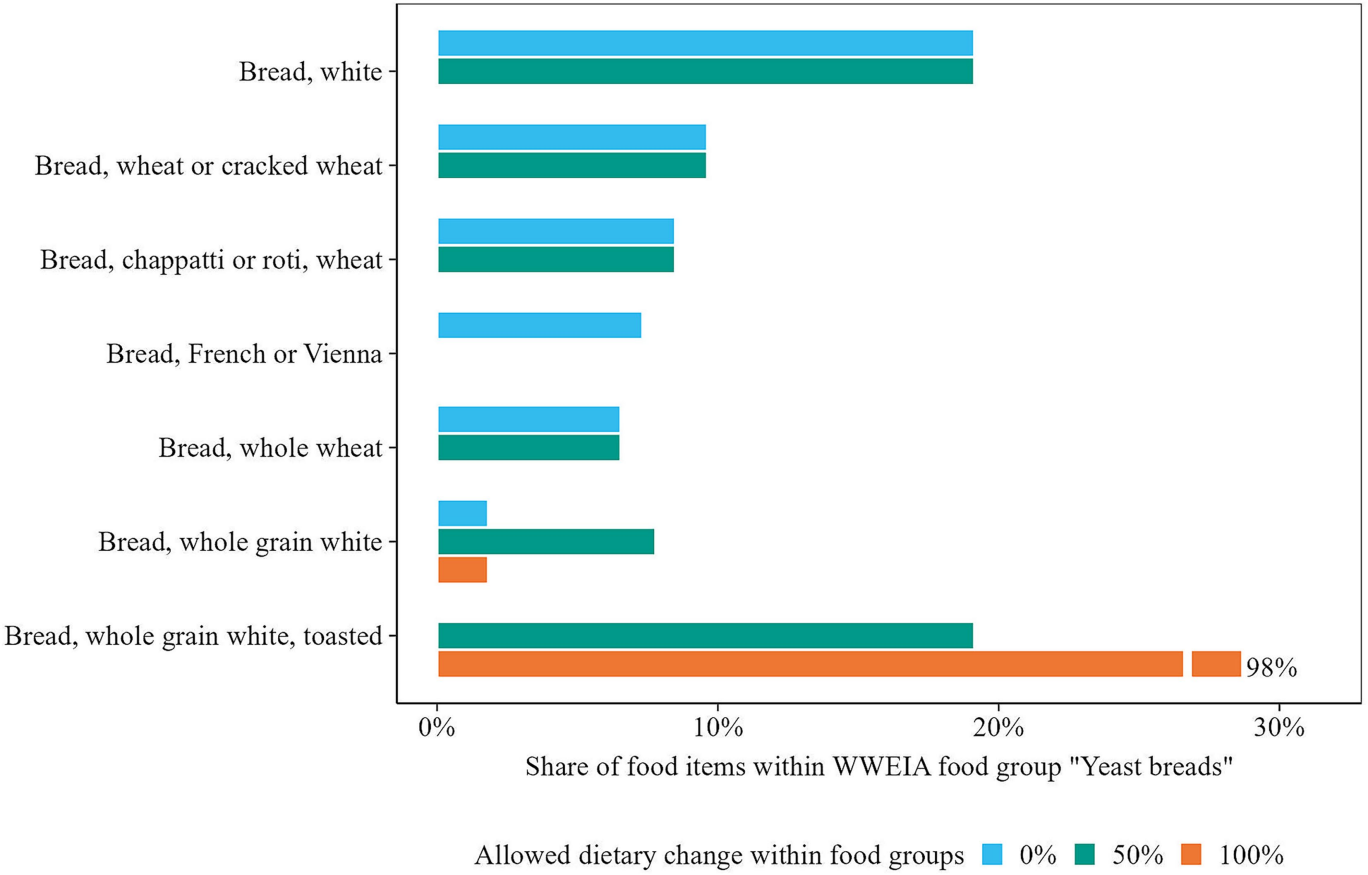
Distribution of foods with the highest share consumed in WWEIA group “Yeast breads” in observed and optimized diets for females. Note that in the scenario where dietary change within food groups is limited to 50%, also no food item can exceed the highest observed quantity within its group.

An example of how within-food-group optimization improved the nutrient content of diets is shown in Figure 6. For females, iron intake substantially increased through the selection of iron-rich food items in the scenario where the allowed change within food groups was 100%. For instance, in the “Yeast breads” group, the model favored “Bread, whole grain white, toasted” (5.4 mg/100 g) over less iron-rich breads like “Bread, white” (3.4 mg/100 g) and “Bread, wheat or cracked wheat” (3.6 mg/100 g), the most consumed breads in the observed diet (Figure 7). In contrast, when dietary change was limited to 50%, a smaller increase in iron was observed, as it was constrained to stay closer to observed consumption patterns.

### 3.2 Lowered nutrient goals

**Figure 8:**
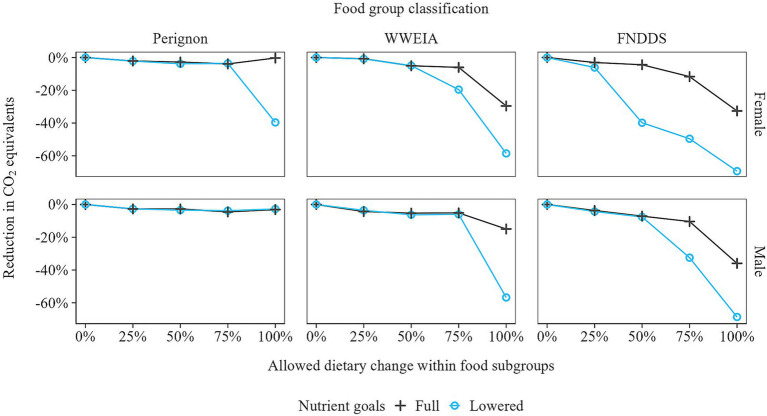
Greenhouse gas emission reductions of the optimized diets, relative to the observed diets, under full and lowered nutrient goals. For the lowered nutrient goals, macronutrient bounds were extended by 20%, and micronutrient goals were lowered from the Recommended Dietary Allowance (RDA) to the Estimated Average Requirements (EAR).

When nutrient goals were lowered by extending macronutrient bounds by 20% and lowering micronutrient goals from the RDA to the EAR, larger reductions in GHGE were achievable (Figure 8). This greater reduction was most evident when 100% of the diet could be adjusted. In this scenario, GHGE reductions exceeding 60% were possible, representing a twofold improvement compared to the full nutrient goals. Larger reductions in GHGE were also observed in the scenario where 75% of the diet could be altered, when the WWEIA and FNDDS classifications were used. However, in the other scenarios, lowering the nutrient goals did not result in a notable GHGE reductions. In these cases, the nutrient goals remained unachieved, leaving limited opportunity for further reductions in GHGE.

**Figure 9:**
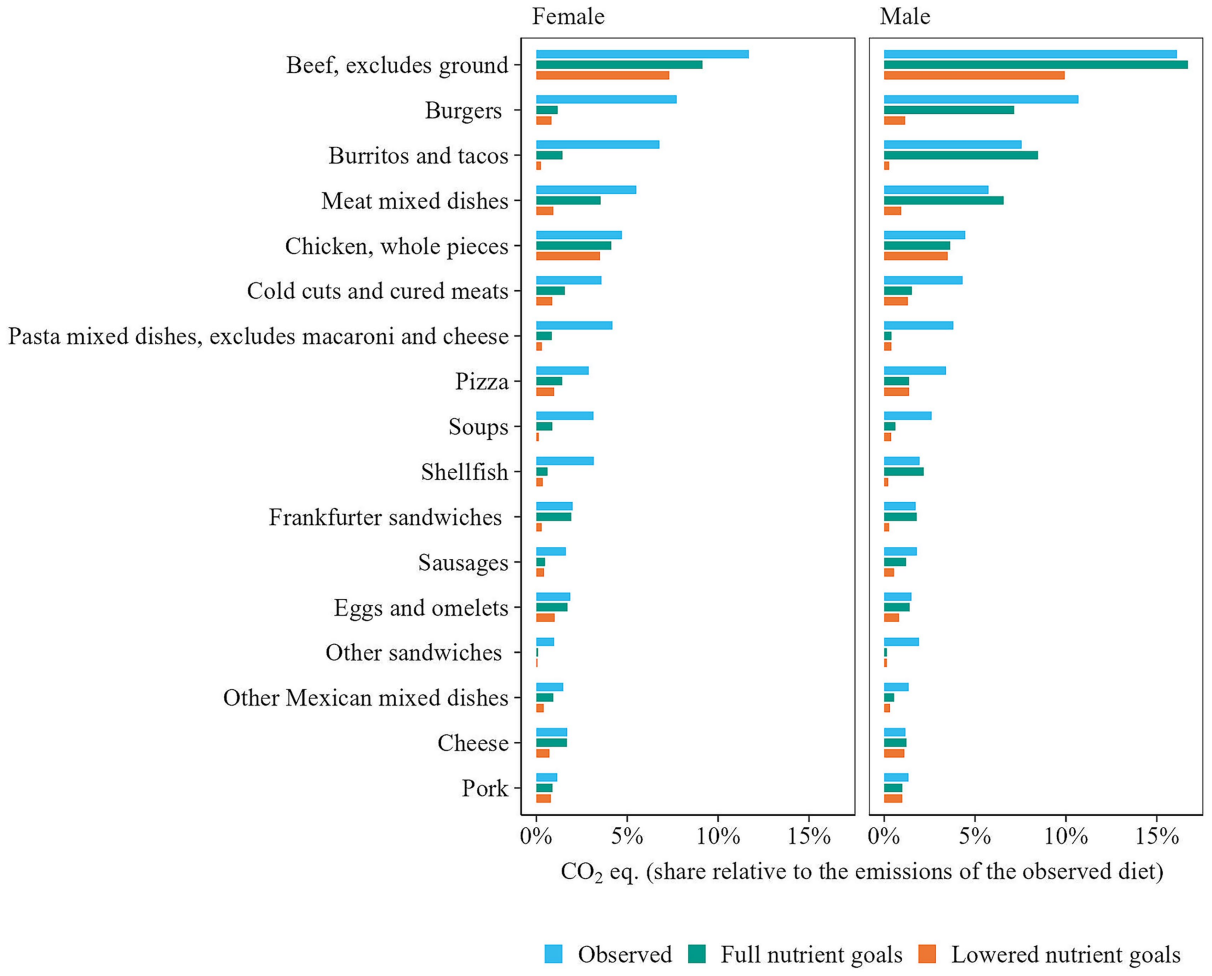
Greenhouse gas emissions from WWEIA food groups (≥3 items) with the highest contributions in observed and optimized diets, under full and lowered nutrient goals. For the lowered nutrient goals, macronutrient bounds were extended by 20%, and micronutrient goals were lowered from the Recommended Dietary Allowance (RDA) to the Estimated Average Requirements (EAR).

Examples of GHGE reductions for a selection of WWEIA food groups are shown in Figure 9. Reductions in groups such as “Burgers” and “Meat mixed dishes” were achieved by replacing beef with turkey or chicken. For groups such as “Burritos and tacos” and “Pasta dishes,” switching to vegetarian options lowered GHGE. Notably, for males, no reductions were observed in groups such as “Beef” or “Meat mixed dishes” when full nutrient goals were applied. This was due to males’ relatively high salt intake, which led the model to favor foods low in sodium over those with low GHGE.

### 3.3 Between-food-group vs. between-and-within-food-group optimization

In the previous sections, we presented improvements in nutritional adequacy and GHGE of diets when optimizing food item quantities within food groups only. In this section, we will examine the dietary changes required to achieve a 30% reduction in GHGE while complying with macro- and micronutrient (RDA) guidelines. We compared two modeling approaches: between-food-group optimization, where only total food group quantities can be adjusted while the distribution of food items within each group remains fixed, as well as between-and-within food-group optimization, where the quantities of individual food items can be adjusted freely (100% allowed dietary change).

**Figure 10:**
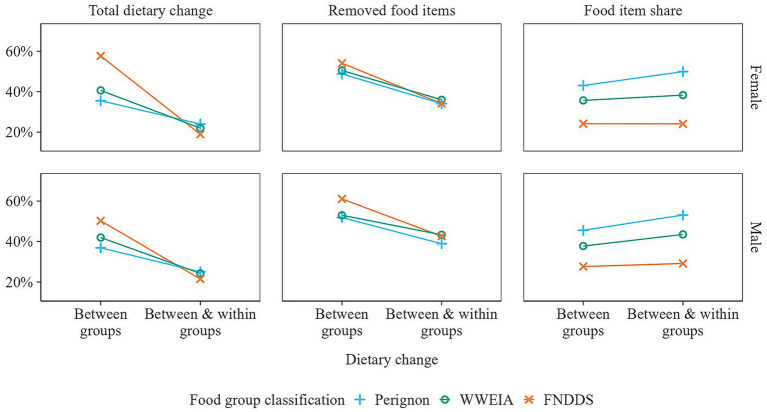
Comparison of between- versus between-and-within-food-group optimization for various acceptability measures. The calculation of total dietary change is detailed in Supplementary Method 1. The percentage of removed food items and the share of food items reflect diet diversity, with food item share being the average share of the most prevalent food item in each food group.

For between-food-group optimization, the total dietary change ranged from 36 to 58%, depending on the food classification used (Figure 10). In contrast, for between-and-within-food-group optimization, the average required dietary change was 23%, which is half of that for between-food-group optimization (44%). Furthermore, the diets obtained by between-and-within-food-group optimization contained a higher number of unique food items. Here, only 38% of food items were removed on average, compared to 53% when applying between-food-group optimization, during which certain food groups were entirely removed. Nonetheless, the latter resulted in more balanced diets, with the average maximum food item share within a food group at 36%, similar to the observed diets, compared to 40% for between-and-within-food-group optimization. Note that the share is relatively higher for food group classifications with fewer food items within each food group.

## 4 Discussion

This study demonstrated that between-and-within-food-group optimization facilitates greater improvements in the nutritional adequacy, environmental sustainability, and acceptability of diets than between-food-group optimization alone. By adjusting food item quantities within food groups only, macro- and micronutrient recommendations could be met while achieving up to a 36% reduction in GHGE. A further reduction in GHGE, exceeding 60%, could be attained by lowering macro- (by 20%) and micronutrient (RDA to EAR) goals. To achieve a 30% reduction in GHGE while complying with macro- and micronutrient (RDA) guidelines, between-and-within-food-group optimization required, on average, only half the total dietary change (23%) compared to between-food-group optimization (44%). This 44% dietary change in U.S. diets for between-food-group optimization aligns with findings by Vieux et al. (9) for France and Finland (45% on average). It is lower than the values reported for the United Kingdom and Sweden (62%) (9) and by Green et al. (6) for the United Kingdom (68%), and exceeds that of Nordman et al. (18) for Denmark (30%) (Table 1). In summary, this study quantified the positive impact of the modeling decision to optimize food item quantities within food groups, as opposed to not doing so, on the nutritional adequacy, environmental sustainability, and acceptability of diets. Note that these results only provide an indication of the potential improvements achieved by optimizing foods within food groups, compared to not doing so. In different study settings, such as when applied to another target population or when additional nutritional constraints are included, the benefits of within-food-group optimization may vary in magnitude. In the remainder of this section, we will also discuss possible drawbacks of within-food-group optimization, as well as briefly address other modeling decisions, including handling missing data and outliers, selecting sustainability metrics, choosing dietary reference values, and quantifying consumer acceptability.

A potential drawback of optimizing food item quantities within food groups is the concern that only a narrow range of the healthiest and most sustainable foods is selected, making modeled diets less diverse and more sensitive to inaccuracies in nutrient (34) and GHGE (35) data. However, rather than avoiding within-food-group optimization altogether, this risk can be managed by applying constraints on food item quantities to help preserve dietary diversity, as demonstrated by our modeling scenarios where the percentage of allowed dietary change within food groups and the maximum quantity of food items were restricted. Additionally, the impact of data inaccuracies can be mitigated through outlier handling techniques, such as winsorization (36).

Furthermore, within-food-group optimization requires GHGE data on food item level, which is not always available. When different data sources do not share the same level of detail, Gazan et al. (8) proposed to aggregate more detailed data (e.g., nutrient composition) to match the least detailed data source (e.g., GHGE). Instead, we recommend modeling at the available levels, where GHGE is optimized at group level and nutrient content at food item level. This approach makes it easier to achieve nutrient goals, thereby requiring less dietary change to achieve the same GHGE reduction. It offers a temporary solution until GHGE data becomes available for a wider range of foods, a need also highlighted by other researchers (22). Alongside GHGE data, it is equally important to collect and model data on other sometimes divergent sustainability mid-point metrics, such as blue water use (or water scarcity), or end-point metrics such as biodiversity, in order to support a more multidimensional approach to designing sustainable diets.

Regarding the modeling decision of which dietary reference values to apply, there may be a trade-off between nutritional adequacy and environmental sustainability. Lowering nutritional goals can allow for greater reductions in GHGE, as shown by Perignon et al. (2) and this study (Figure 8). A question that remains is the extent to which improving sustainability may come at the expense of nutritional adequacy? For example, the Recommended Daily Allowances (RDA) serve as guidelines for micronutrient intake to meet the needs of 97.5% of the population and are often used in individual diet counseling. Many individuals, however, can remain healthy with lower intake levels, such as the Estimated Average Requirements (EAR), which meet the needs of 50% of the population (37, 38). Alternatively, increased use of dietary supplements and food fortification could help improve the sustainability of diets (39, 40).

Among all modeling decisions, determining how to quantify consumer acceptability may be the most technically challenging. In this study, we assumed that diets with greater diversity and fewer changes from the observed diet would be more acceptable. However, there is still limited understanding of which types of dietary change are most acceptable to consumers (3, 4). For instance, are small adjustments across a variety of foods more acceptable than large changes to a few? Is it easier for consumers to adapt to the addition of new foods or to the elimination of familiar ones? Are modifications within food groups preferred over changes between groups? Although modifications within food groups may be preferred because the alternatives are likely more similar in taste (41), a potential downside is that dietary advice at individual food level can be more challenging to recall and follow than general food group recommendations (42). As for food diversity, its association with acceptability is not necessarily linear. For example, a diet with many different food items could become less acceptable if the preparation of such meals is deemed too effortful by consumers. Conversely, diets with less variety, like having the same breakfast every day, may be acceptable for consumers who prefer routine. Further research is needed to address these questions to ensure optimized diets are not only healthy and sustainable but also acceptable to individuals. This involves both understanding the behavioral drivers of consumer acceptance as well as developing methods to quantify and integrate those drivers into diet modeling.

## 5 Conclusion

This study demonstrated that within-food-group optimization increases opportunities to improve the nutritional adequacy, sustainability, and acceptability of diets. As such, we recommend optimizing both between and within food groups.

## Data Availability

Publicly available datasets were analyzed in this study. This data can be found at: https://wwwn.cdc.gov/nchs/nhanes/continuousnhanes/default.aspx?BeginYear=2017. Additional data sources can be found in the Methods section.
