# Supplementary material for: Within-food-group optimization improves nutritional adequacy, sustainability, and acceptability of modeled diets
Source: Front Nutr. 2025 Sep 30;12:1648055. doi: 10.3389/fnut.2025.1648055 (PMC12518068; doi:10.3389/fnut.2025.1648055)
Supplement: Supplementary file 1 [file Data_Sheet_1.zip › Supplementary Methods.PDF]

Supplementary Material

1 Calculation of dietary change

Among studies, different metrics are used to calculate dietary change. For example, Vieux et al. (1) reported the mean absolute quantity variation (%) across 151 food items, referred to here as food groups, using the following equation:

$$\frac{1}{G}\sum_{g=1}^{G}\frac{\left|Q_g^{optimized}-Q_g^{observed}\right|}{Q_g^{observed}}$$

where Q_g represents the quantity of food group g in the observed and optimized diets, and G is the total number of food groups.

Green et al. (2) displayed the sum of squared percentage deviations (%) for 148 food groups:

$$\sum_{g=1}^{G}\left(\frac{Q_g^{optimized}-Q_g^{observed}}{Q_g^{observed}}\right)^2$$

In this study, we report the total dietary change (%) across 2,734 food items i:

$$\frac{\sum_i\left|Q_i^{optimized}-Q_i^{observed}\right|}{\sum_i Q_i^{observed}}$$

This metric was chosen because it is more stable than Vieux et al.'s (1), where small denominators can cause large percentage differences when calculated at food item level. Additionally, it is easier to interpret than Green et al.'s (2) metric, as squared changes can be less intuitive.

To compare our modeling results with the studies of Vieux et al. (1), Green et al. (2), Reynolds et al. (3), Nordman et al. (4), and Kesse-Guyot et al. (5) we calculated total dietary change for their studies using the food group quantities reported in their manuscript or supplementary materials:

$$\frac{\sum_g \left| Q_g^{optimized} - Q_g^{observed} \right|}{\sum_g Q_g^{observed}}$$

These outcomes are reported in the introduction. For the studies by Perignon et al. (6), Horgan et al. (7), Rocabois (8), and Heerschop et al. (9), this was not possible, so we reported the outcomes as presented by the original authors.

2 Custom food group classification

Following the methodology of Perignon et al. (6) and Vieux et al. (10), we began by selecting the most frequently consumed foods in each of the 153 WWEIA food groups (11). Selection was based on ranking foods by the number of respondents who consumed them, with the most popular food ranked first. Foods were included if their rank fell between 1 and a threshold calculated as 10% of the total number of items in the food group. In this way, a greater number of foods were selected for groups containing more items. For example, the WWEIA group ‘apple juice’ contains only three food items. Since 10% of 3 equals 0.3, which rounds up to 1, only the item ranked first was selected. Similarly, in the larger group ‘beans, peas, legumes’, which contains 61 food items, the threshold allowed for the selection of items ranked between 1 and 7. Overall, this approach led to the selection of 345 foods, which were used as custom groups for assigning the remaining food items to the foods with most similar nutrient profiles, based on their scaled Euclidean distance. The nutrients considered in this process are listed in Supplementary Table 4. Of the 345 custom groups formed, 32 contained a single food item, while the largest group consisted of 45 items.

3 Diet model

3.1 Notation

Supplementary Table 1. Indices, parameters, and variables.

Indices	
i	Food item i = 1 .. I
g	Food group g = 1 .. G
n	Nutrient n = 1 .. N
n.macro	Subset of macronutrients
n.macro.e	Subset of macronutrients where bounds are expressed as percentage of energy intake
n.macro.w	Subset of macronutrients where bounds are expressed in weight
n.micro	Subset of micronutrients

Parameters	
ε	Arbitrary small value
b_{ig}	Binary value indicating food item i belongs to food group g (yes:1 ; no:0)
c_{in}	Nutrient content for food item i and nutrient n
e_i	Emission for food item i
e^f	Emission of food items that may not be substituted (fixed)
e_g^{fg}	Average emission for food group g.
ear_n	Estimated Average Requirement (EAR) for each micronutrient n
f^b	Factor to extend macronutrient bounds
f^d	Fraction of allowed quantity deviation per food group
f^e	Energy conversion factor
f^r	Greenhouse gas emission (GHGE) reduction factor
lb_n	Lower bound on intake level for each macronutrient n
o_i	Observed food intake for food items i
o^{en}	Observed energy intake
o^{em}	Observed emission
o_g^{fg}	Observed food intake for food group g
$o_g^{fg.max}$	Maximum observed food intake within food group g
$o_{ig}^{fg.sh}$	Observed food intake share of food item i within food group g
rda_n	Recommended Daily Allowance (RDA) for each micronutrient n
t_n^f	Nutrient intake of food items that may not be substituted (fixed) for nutrient n
ub_n	Upper bound on intake level for each macronutrient n
ul_n	Tolerable upper intake level for each micronutrient n

Variables	
D_n^{ear}	Normalized deviation below EAR for micronutrient n
$D^{max.ear}$	Maximum micronutrient deviation from the EAR
$D^{max.macro}$	Maximum macronutrient deviation from dietary guidelines
$D^{max.q}$	Maximum absolute deviation from observed quantity
$D^{max.rda}$	Maximum micronutrient deviation from the RDA
D_n^{lb}	Normalized deviation below lower bound for macronutrient n
D_i^{q}	Absolute deviation from observed quantity for food item i
D_i^{q-}	Negative deviation from observed quantity for food item i
D_i^{q+}	Positive deviation from observed quantity for food item i
D_n^{rda}	Normalized deviation below RDA for micronutrient n
$D^{sum.ear}$	Sum of micronutrient deviations from the EAR
$D^{sum.macro}$	Sum of macronutrient deviations from dietary guidelines
$D^{q.sum}$	Sum of absolute deviations from observed quantity
$D^{sum.rda}$	Sum of micronutrient deviations from the RDA
D_n^{ub}	Deviation above upper bound for macronutrient n
E	Emission
Q_i	Quantity for food item i
Q_g^{fg}	Quantity for food group g
T_n	Nutrient intake for nutrient n

3.2 Base model

The diet model optimizes nutrient and greenhouse gas emission (GHGE) goals by changing food quantities between and within food groups. Different strategies for achieving this are explored through various modeling experiments. This section outlines the constraints of the base model, which are applied across all experiments (Supplementary Table 2). The following section discusses the additional constraints and objective functions specific to each modeling experiment (Supplementary Table 3).

Constraints 1 and 2 measure the absolute change in quantity relative to observed consumption. Constraint 3 then calculates the total deviation in food quantities compared to the observed diet, and Constraint 4 determines the maximum deviation, normalized by observed food group quantities. Food group quantities are calculated with Constraint 5.

Next, the nutrient intake of the optimized diet is calculated (Constraint 6), comprising two components: intake from optimized food items and intake from foods excluded from optimization. Micronutrient intake is constrained by the tolerable upper intake levels (Constraint 7), and goals are established according to the Estimated Average Requirement (EAR) (Constraint 8) and Recommended Daily Allowances (RDA) (Constraint 9) (Supplementary Table 4). Deviations from these goals are normalized to a 0–1 scale for comparison. Constraints 10 and 11 then evaluate the maximum deviations below the EAR and RDA, and Constraints 12 and 13 calculate the total sum of these deviations.

Furthermore, Constraint 14 guarantees that the optimized diet maintains an energy intake similar to the observed diet. Constraints 15–18 set the macronutrient goals in alignment with dietary guidelines, distinguishing between goals expressed as energy percentages (Constraints 15–16) and those expressed in weight (Constraints 17–18). To ensure model linearity for the energy percentage goals, deviations are normalized using the energy intake of the observed diet.

The macronutrient bounds can be relaxed by a factor to make them easier to meet. Constraints 19 and 20 calculate the maximum deviation and total deviations for macronutrient intake.

GHGE of the observed diet are calculated using Constraint 21. As with nutrient intake, emissions are divided into two components: those from optimized food items and those from foods excluded from optimization. Constraint 22 ensures that the emissions from the optimized diet are less than or equal to those of the observed diet.

Finally, Constraints 23–26 establish the variable domains.

Supplementary Table 2. Constraints of base diet model.

Constraints		
$Q_i - o_i = D_i^{q+} - D_i^{q-}$	∀ i	(1)
$D_i^q = D_i^{q+} + D_i^{q-}$	∀ i	(2)
$D^{sum.q} = \sum_i D_i^q$		(3)
$D^{max.q} \geq {D_i^q \cdot b_{ig}}/{o_g^{fg}}$	∀ i, g	(4)
$Q_g^{fg} = \sum_i (Q_i \cdot b_{ig})$	∀ g	(5)
$T_n = \sum_i (Q_i \cdot c_{in}) + t_n^f$	∀ n	(6)
$T_n \leq ul_n$	∀ n.micro	(7)
$T_n + ear_n \cdot D_n^{ear} \geq ear_n$	∀ n.micro	(8)
$T_n + rda_n \cdot D_n^{rda} \geq rda_n$	∀ n.micro	(9)
$D^{max.ear} \geq D_n^{ear}$	∀ n.micro	(10)
$D^{max.rda} \geq D_n^{rda}$	∀ n.micro	(11)
$D^{sum.ear} = \sum_n D_n^{ear}$		(12)
$D^{sum.rda} = \sum_n D_n^{rda}$		(13)
$T_{Energy} = o^{en}$		(14)
$T_n + D_n^{lb} \cdot lb_n \cdot (1 - f^b) \cdot {o^{en}}/{f^e} \geq lb_n \cdot (1 - f^b) \cdot {T_{Energy}}/{f^e}$	∀ n.macro.e	(15)
$T_n - D_n^{ub} \cdot ub_n \cdot (1 + f^b) \cdot {o^{en}}/{f^e} \leq ub_n \cdot (1 + f^b) \cdot {T_{Energy}}/{f^e}$	∀ n.macro.e	(16)
$T_n + D_n^{lb} \cdot lb_n \cdot (1 - f^b) \geq lb_n \cdot (1 - f^b)$	∀ n.macro.w	(17)
$T_n - D_n^{ub} \cdot ub_n \cdot (1 + f^b) \geq ub_n \cdot (1 + f^b)$	∀ n.macro.w	(18)
$D^{max.macro} \geq D_n^{lb} + D_n^{ub}$	∀ n.macro	(19)
$D^{sum.macro} = \sum_n (D_n^{lb} + D_n^{ub})$		(20)
$E = \sum_i (Q_i \cdot e_i) + e^f$		(21)
$E \leq o^{em}$		(22)
$D^{max.ear}, D^{max.macro}, D^{max.rda}, D^{max.q}, D^{sum.ear}, D^{sum.macro}, D^{sum.rda}, D^{sum.q}, E \in \mathbb{R}_{\geq 0}$		(23)
$D_n^{ear}, D_n^{lb}, D_n^{rda}, D_n^{ub}, T_n \in \mathbb{R}_{\geq 0}$	∀ n	(24)
$D_i^q, D_i^{q-}, D_i^{q+}, Q_i \in \mathbb{R}_{\geq 0}$	∀ i	(25)
$Q_g^{fg} \in \mathbb{R}_{\geq 0}$	∀ g	(26)

3.3 Modeling experiments

Within-food-group optimization

In the first modeling experiment, we examine the potential to improve the nutritional adequacy and greenhouse gas emissions (GHGE) of observed diets by adjusting food item quantities within food groups only. This means that the total quantity of each food group remains fixed, as in the observed diet (Constraint 27, Supplementary Table 3). We evaluate this under varying levels of allowed dietary change. In the most restrictive scenario (0% change), the diet remains identical to the observed diet. In the least restrictive scenario (100% change), all food item quantities within each food group may be freely adjusted. For intermediate scenarios, we constrain the absolute change within each food group to x% (f^d) or less (Constraint 28'). Additionally, no food item may exceed the highest quantity observed within its respective food group (Constraint 29'). In the objective function, different goals are weighted, with the highest weight assigned to minimizing the largest deviation from recommended macro- and micronutrient intake levels (RDA), followed by the total sum of nutrient deviations, then GHGE reduction, and finally, minimizing overall dietary change (Constraint 30).

Lowered nutrient goals

In the second modeling experiment, which builds on the first, we investigate to what extent lowering nutrient goals can further reduce GHGE. Here, macronutrient bounds are relaxed by a specified factor (f^b, Constraints 15–18, Supplementary Table 2), and in the objective function, the micronutrient goals are lowered from the RDA to the EAR (Constraint 31, Supplementary Table 3).

Between-food-group vs. Between-and-within-food-group optimization

In the last modeling experiment, we investigate the minimal amount of dietary change required to achieve specified nutrient (Constraints 32–33, Supplementary Table 3) and GHGE (Constraint 34) goals. We compare the required dietary change under two scenarios: one where only total food group quantities may be adjusted—keeping the composition of food items within each group unchanged (Constraint 35')—and another where food item quantities can be changed without restriction. Additionally, we compare the effects of calculating GHGE at the food group level (Constraint 36') versus the food item level (Constraint 21, Supplementary Table 2). In the objective function, the highest priority is given to minimizing dietary change, followed by further reducing GHGE (Constraint 37, Supplementary Table 3).

The diet models were run in Python 3.11.4 with Gurobi 11.0.1, while all other data preparation and analyses were conducted in R 4.2.1.

Supplementary Table 3. Additional constraints and objective function for each modeling experiment.

Within-food-group optimization

$Q_g^{fg} = o_g^{fg}$	$\forall$ g	(27)
$\sum_i \left(D_i^q \cdot b_{ig}\right) \leq f^d \cdot o_g^{fg}$	$\forall$ g	(28’)
$\sum_i \left(D_i^q \cdot b_{ig}\right) \leq o_g^{fg.max}$	$\forall$ i, g	(29’)
$min\,\{D^{max.macro} + D^{max.rda} + \varepsilon_1\,(D^{sum.macro} + D^{sum.rda}) + \varepsilon_2 \cdot E + \varepsilon_3 \cdot D^{sum.q}\}$		(30)

Lowered nutrient goals

$min\,\{D^{max.macro} + D^{max.ear} + \varepsilon_1\,(D^{sum.macro} + D^{sum.ear}) + \varepsilon_2 \cdot E + \varepsilon_3 \cdot (D^{max.rda} + D^{sum.rda}) + \varepsilon_4 \cdot D^{sum.q}\}$	(31)

Between-food-group vs. Between-and-within-food-group optimization

$D^{max.macro} \leq 0$		(32)
$D^{max.rda} \leq 0$		(33)
$1 - E/_{O^{em}} \geq f^r$		(34)
$Q_i = \sum_g \left(Q_g^{fg} \cdot o_{ig}^{fg.sh}\right)$	$\forall$ i	(35’)
$E = \sum_g \sum_i \left(Q_i \cdot b_{ig} \cdot e_g^{fg}\right) + e^f$		(36’)
$min\,\{D^{max.q} + 0.001 \cdot D^{sum.q} + \varepsilon \cdot E\}$		(37)

3.4 Parameter input

Supplementary Table 4. Dietary reference intakes (women and men 19-30y) for nutrients considered in the diet models (12, 13)[1].

		LB		UB	
Macronutrient	Unit	Female	Male	Female	Male
Energy[2]	kcal/day	1800	2400	2400	3000
Protein	E%	10	10	35	35
Total carbohydrates	E%	45	45	65	65
Dietary fibre	g	25	38	-	-
Total fat	E%	20	20	35	35
Total saturated fatty acids	E%	-	-	10	10
Sodium	mg	-	-	2300	2300

		EAR		RDA		
Micronutrient[3]	Unit	Female	Male	Female	Male	UL
Calcium	mg	800	800	1000	1000	2500
Copper	mg	0.7	0.7	0.9	0.9	10
Iron	mg	8.1	6	18	8	45
Magnesium	mg	255	330	310	400	-
Selenium	µg	45	45	55	55	400
Zinc	mg	6.8	9.4	8	11	40
Vitamin A	µg	500	625	700	900	3000
Vitamin B1	mg	0.9	1	1.1	1.2	-
Vitamin B2	mg	0.9	1.1	1.1	1.3	-
Vitamin B3	mg	11	12	14	16	-
Vitamin B6	mg	1.1	1.1	1.3	1.3	100
Vitamin B9	µg	320	320	400	400	1000
Vitamin B12	µg	2	2	2.4	2.4	-
Vitamin C	mg	60	75	75	90	2000
Vitamin E	mg	12	12	15	15	-

[1] Abbreviations: LB, Lower Bound; UB, Upper Bound; EAR, Estimated Average Requirement; RDA, Recommended Daily Allowance; UL, tolerable Upper Level.
[2] Energy reference intakes for a sedentary (LB) and active (UB) lifestyle.
[3] The diet model does not optimize vitamin D intake because guidelines are based on both food intake and sunlight exposure.

4 References

1. Vieux, F., et al. (2018). Dietary changes needed to improve diet sustainability: are they similar across Europe? *European journal of clinical nutrition*, *72*(7), 951-960.
2. Green, R., et al. (2015). The potential to reduce greenhouse gas emissions in the UK through healthy and realistic dietary change. *Climatic Change*, *129*, 253-265.
3. Reynolds, C.J., et al. (2019). Healthy and sustainable diets that meet greenhouse gas emission reduction targets and are affordable for different income groups in the UK. *Public health nutrition*, *22*(8), 1503-1517.
4. Nordman, M., et al. (2024). Low-carbon diets across diverse dietary patterns: Addressing population heterogeneity under constrained optimization. *Science of the Total Environment, 953*, 176155.
5. Kesse-Guyot, E., et al. (2025). To be climate-friendly, food-based dietary guidelines must include limits on total meat consumption–modeling from the case of France. *International Journal of Behavioral Nutrition and Physical Activity*, *22*(1), 95.
6. Perignon, M., et al. (2016). How low can dietary greenhouse gas emissions be reduced without impairing nutritional adequacy, affordability and acceptability of the diet? A modelling study to guide sustainable food choices. *Public health nutrition*, *19*(14), 2662-2674.
7. Horgan, G.W., et al. (2016). Achieving dietary recommendations and reducing greenhouse gas emissions: modelling diets to minimise the change from current intakes. *International Journal of Behavioral Nutrition and Physical Activity*, *13*, 1-11.
8. Rocabois, A., et al. (2022). Diet optimization for sustainability: INDIGOO, an innovative multilevel model combining individual and population objectives. *Sustainability*, *14*(19), 12667.
9. Heerschop, S.N., et al. (2023). Shifting towards optimized healthy and sustainable Dutch diets: impact on protein quality. *European Journal of Nutrition*, *62*(5), 2115-2128.
10. Vieux, F., et al. (2013). High nutritional quality is not associated with low greenhouse gas emissions in self-selected diets of French adults. *The American journal of clinical nutrition*, *97*(3), 569-583.
11. U.S. Department of Agriculture. Agricultural Research Service. (2020). *USDA Food and Nutrient Database for Dietary Studies 2013-2018*.
12. National Institutes of Health. *Nutrient Recommendations and Databases*. 2024; Available from: https://ods.od.nih.gov/HealthInformation/nutrientrecommendations.aspx.
13. U.S. Department of Agriculture and U.S. Department of Health and Human Services. (December 2020). Dietary Guidelines for Americans, 2020-2025.
